# Immunoediting: evidence of the multifaceted role of the immune system in self-metastatic tumor growth

**DOI:** 10.1186/1742-4682-9-31

**Published:** 2012-07-28

**Authors:** Heiko Enderling, Lynn Hlatky, Philip Hahnfeldt

**Affiliations:** 1 Center of Cancer Systems Biology, Steward St. Elizabeth’s Medical Center, Tufts University School of Medicine, 736 Cambridge Street, Boston, MA, 02135, USA

## Abstract

**Background:**

The role of the immune system in tumor progression has been a subject for discussion for many decades. Numerous studies suggest that a low immune response might be beneficial, if not necessary, for tumor growth, and only a strong immune response can counter tumor growth and thus inhibit progression.

**Methods:**

We implement a cellular automaton model previously described that captures the dynamical interactions between the cancer stem and non-stem cell populations of a tumor through a process of self-metastasis. By overlaying on this model the diffusion of immune reactants into the tumor from a peripheral source to target cells, we simulate the process of immune-system-induced cell kill on tumor progression.

**Results:**

A low cytotoxic immune reaction continuously kills cancer cells and, although at a low rate, thereby causes the liberation of space-constrained cancer stem cells to drive self-metastatic progression and continued tumor growth. With increasing immune system strength, however, tumor growth peaks, and then eventually falls below the intrinsic tumor sizes observed without an immune response. With this increasing immune response the number and proportion of cancer stem cells monotonically increases, implicating an additional unexpected consequence, that of cancer stem cell selection, to the immune response.

**Conclusions:**

Cancer stem cells and immune cytotoxicity alone are sufficient to explain the three-step “immunoediting” concept – the modulation of tumor growth through inhibition, selection and promotion.

## Background

**Figure 1:**
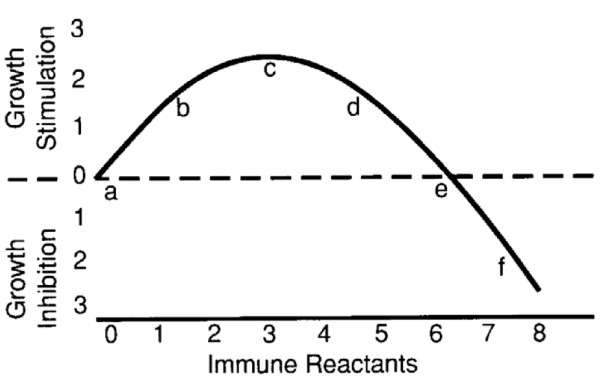
**Dual effect of the immune response in premalignant tumor progression.** A small number of immune reactants paradoxically stimulate growth, whereas a large immune response inhibits tumor progression (reproduced from [8]).

The immune system in mammals is responsible for elimination of damaged cells. The development of tumors is always associated with an immune response [1]. Complete activation of the adaptive immune system might result in complete tumor eradication, but tumor progression and clinical manifestation has demonstrated the ability of tumor cells to escape immunosurveillance, despite efficient immune responses. In fact, a massive influx of activated infiltrating immune cells is correlated with a poor patient prognosis, fueling the hypothesis that an immune reaction may under some circumstances be tumor-promoting [1]. The potential for a tumor-promoting action by the immune system was proposed some time ago [2], but the actual mechanisms are still the subject of debate. We do know that infiltrating macrophages and mast cells can regulate tumor cell proliferation and cell death, and that chronic inflammation can skew the dynamics in favor of tumor growth [1]. More recently it has been hypothesized that the immune system can keep the tumor in a somewhat dormant state, but over time select for more aggressive variants with reduced immunogenicity [3]. This process, often referred to as immunoediting or tumor sculpting, may occur continuously and has major effects early in tumor progression [4]. Conceptually, the process is comparable to the enrichment of radioresistant and chemoresistant neoplastic clones that takes place as part of tumor evolution in the face of treatment by radiation and drugs [5]. The immune system can likewise exacerbate this natural evolutionary process, eliminating sensitive sells while yielding enrichment of immunoresistant tumor variants [6]. Recent evidence has emerged that cancer stem cells can selectively escape the cytotoxic action of immune system killer cells and thus become enriched during an immune response [7]. This raises the prospect that the efficiency of the immune system in eradicating the tumor could be dependent on the ratio of immune reactants to tumor cells. Supporting this idea, a low immune reaction has been shown to accelerate tumor growth, whereas a large numbers of immune reactants inhibit progression [2,8-10] (Figure 1).

Tumor growth dynamics are usually marked by the defining features of immunoediting; initial growth amidst productive immune response, an equilibrium state where tumor growth and suppression by immune response are more or less in balance, and malignant progression, as tumor subpopulations selected for immune resistance or evasion during the previous phase drive tumor expansion [1,4]. The selection of tumor cells resistant to infiltrating immune cells might explain the strong correlation between number of tumor-associated macrophages and poor prognosis [11]. The tumor-promoting effect of macrophages and the immune system in general has been attributed to second-order events such as production of angiogenic factors and matrix metalloproteinases (MMPs), because the primary cytotoxic cell killing is intuitively tumor-inhibiting [11]. However, it has been shown recently that cell kill might paradoxically benefit tumor progression in heterogeneous tumors [12] and in particular, that a sufficient source for this heterogeneity may lie in the tumor-intrinsic interactions between cancer stem cell and non-stem cell fractions that give rise to a ‘self-metastatic’ phenotype [13,14]. Here we present a model of self-metastatic tumor growth subject to immune action, and show in this setting that the basic cytotoxic function of the immune system alone can reproduce the experimentally- and clinically-observed multifaceted features of immunoediting – elimination, equilibrium, and escape.

## Methods

**Figure 2:**
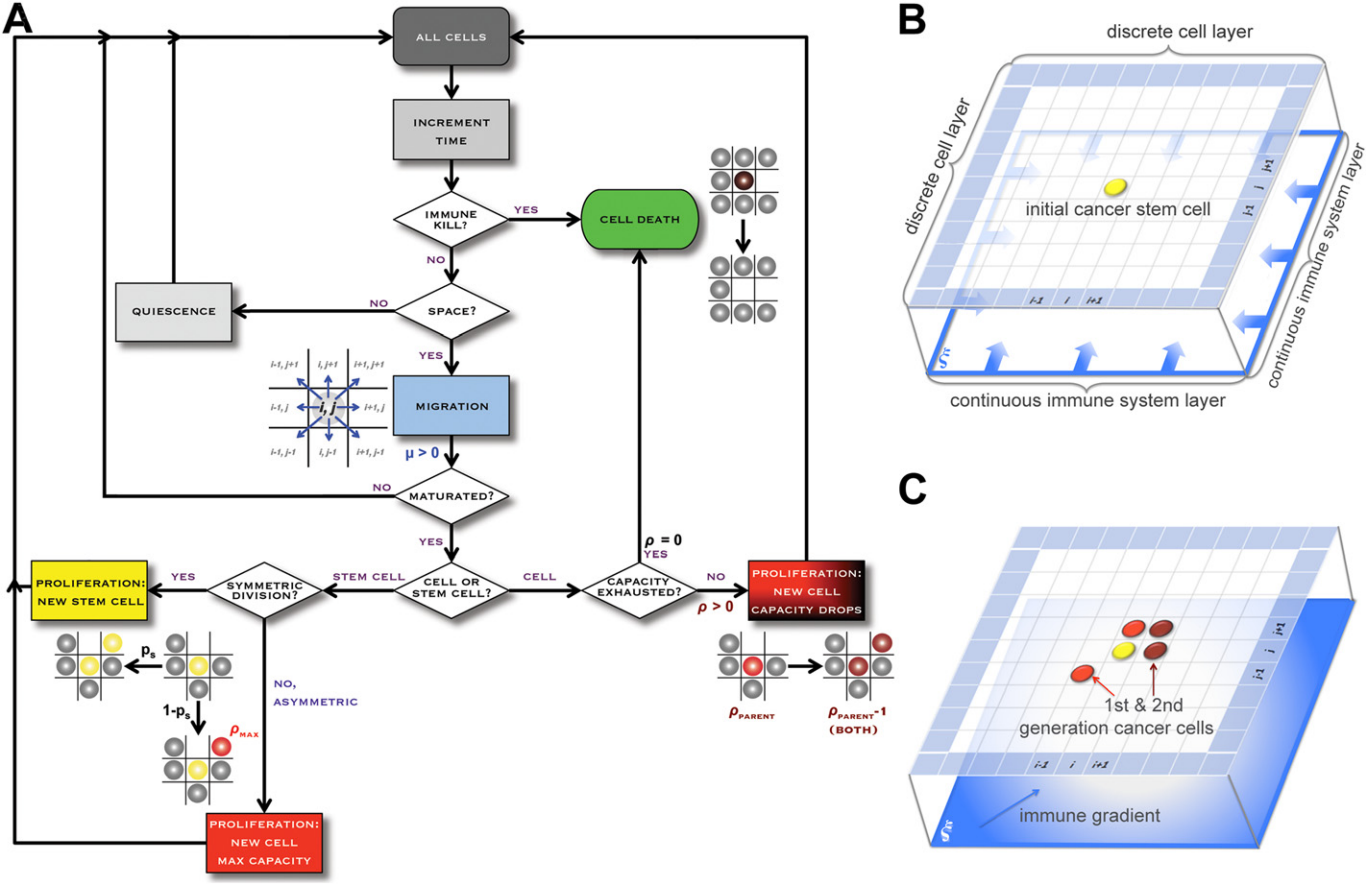
**Schematic of the hybrid model setup. A**) Simulation flowchart. **B**) Cancer cells live on a discrete square lattice with initially a single cancer stem being located in the center of the domain. Immune reactant concentration is modeled as a continuum with sources located at domain boundaries. **C**) A tumor population forms and immune reactants diffuse into the domain.

Various mathematical models have been utilized previously to address different phases of the tumor-immune system interaction, including mechanisms of immunoediting [15-25]. None of the models, however, discuss the ramifications of a tumor being comprised of a heterogeneous population of cancer stem cells and non-stem cancer cells. Here we extend an established cellular automaton model of tumor growth that considers interactions between cancer stem cells and non-stem progenitor cells [13,26-29] to study the impact of the immune system on tumor growth dynamics and tumor composition. We introduce a hybrid discrete-continuum model framework [30,31] where individual cells live on a discrete lattice and the concentration of immune reactants is modeled as a diffusing continuum (Figure 2). The two layers are connected such that cancer cells experience the local concentration of immune reactants, triggering cell death in proportion. For the discrete cell model we assume that cancer stem cells are immortal, i.e. their probability of cell death is α = 0, and have an unlimited proliferative capacity ρ, i.e. ρ=infinity. In contrast, non-stem cancer cells, the more committed offspring of cancer stem cells, can only divide a finite number of times ρ_max_, after which they become unviable and die. Throughout this study we assumed ρ_max_ = 10, in line with previous observations of fast tumor growth [26]. With every cell division, cancer stem cells can either divide symmetrically with probability p_s_ to produce two daughter cancer stem cells, or asymmetrically with probability 1-p_s_ to produce a cancer stem cell and a non-stem progenitor cancer cell. We set p_s_ = 0.01 (i.e., 1%) to reflect the low frequency of cancer stem cells reported in the literature [32]. To initiate the simulation of tumor growth and immune response, we seed single cancer stem cells in the center of a computational domain of 350 × 350 grid points, representing a square lattice of 3,500 μm × 3500 μm subdivided into 100 μm^2^ units that can hold at the most one cell at any time. Cells have a random motility μ (in units of cell widths per day) and accordingly, are considered for migration to an adjacent unit every 1/μ days. In line with previous studies we set μ = 15 (i.e., 15 cell widths or ≈150 μm day^-1^) [13]. Cells can divide after reaching ‘maturation’, which for simplicity is attempted every 1 day. When it is time to attempt migration or division, the moving cell, or the progeny of the dividing cell, will be randomly assigned to an adjacent vacant unit. If there is no adjacent vacant unit the movement (or division) does not take place. Accordingly, a cell that is completely surrounded by other cells is forced to become quiescent, and migration and proliferation are only possible once adjacent lattice points become vacant again. For simplicity we ignore tumor cell interaction with host cells in the immediate tumor microenvironment, as well as density-dependent modulation of cellular fates [33].

The concentration of immune reactants *c* is modeled as a diffusing continuum

(1) $$\frac{\partial c}{\partial t}=\overset{\text{diffusion}}{\overset{︷}{d_{c}\nabla^{2}c}}-\overset{\text{decay}}{\overset{︷}{\beta c}}\text{,}$$

where *d*_*c*_ = 10^-9^ cm^2^s^-1^ is the diffusion coefficient comparable to cell motility estimated experimentally [34] and *β* is the decay rate of immune reactants. We further assume immune system sources to be located at the domain boundary, and as the tumor grows, immune agents are produced with either (*i*) a constant strength ξ, reflecting the probability of cell kill at the boundary ∂Ω of the total domain Ω, from which it follows that 0 ≤ ξ ≤ 1, equated with the (presumed fixed) boundary concentration, i.e.,

(2) $$c|{}_{\partial \Omega }=\xi \text{,}$$

or (*ii*) dependent on the tumor size in response to a growing cell population, as later described. The probability *α* of immune reactant-induced death for a non-stem cancer cell at position (*xy*) at time *t* is equated to the immune reactant concentration at this position at that time, i.e., *α=c*(*xyt*). In line with recent literature we assume cancer stem cells evade the immune response [6,7,35,36]. A schematic of the cell dynamics and the hybrid two-layer architecture is shown in Figure 2.

## Results

### Dual effect of the immune system

**Figure 3:**
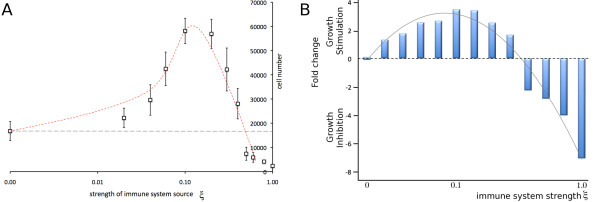
**Dual immune-reactant-dependent growth dynamics. A**) Non-monotonic variation in cell number over immune system strength. **B**) Immune system strength dependent growth stimulation and growth inhibition (cf. Figure 1).

We simulate tumor growth from a single cancer stem cell for t = 730 days and report the averages of 10 independent simulations. For simplicity we ignore immune reactant decay (β = 0). Without an immune response, i.e. when ξ = 0, the tumor has 16,749 cells, of which 15 cells are cancer stem cells (0.09%). Increasing the immune reactant source strength to ξ = 0.04 and ξ = 0.1 yields bigger tumors (29,596 and 58,134 cells, respectively) with more cancer stem cells and greater stem cell ratios (44 (0.15%) and 219 (0.38%)). When the immune system strength is further increased, a reverse effect and decreasing tumor cell numbers can be seen. Immune responses of ξ = 0.4 and ξ = 1 lead to tumors containing 25,502 cells (1,011 cancer stem cells; 3.99%) and 2,365 cells (1177 cancer stem cells, 49.8%) (summarized in Table 1). The tumor size as a function of the immune system strength is shown in Figure 3. When plotted as a fold change with respect to the tumor grown without an immune response, i.e. ξ = 0, the curve resembles the initial growth stimulation and final inhibition as a function of immune reactant concentration predicted by Prehn [2,8-10] (cf. Figure 1).

**Table 1:** Simulation statistics

**ξ**	**Total population**	**Cancer stem cells**	**Cancer stem cell ratio (%)**
0	16,749	15	0.09
0.04	29,596	44	0.15
0.1	58,134	219	0.38
0.4	25,502	1,018	3.99
1	2,365	1,177	49.8

Average statistics for n = 10 independent runs each after t = 730 days.

### Self-metastastic morphology and immune selection

**Figure 4:**
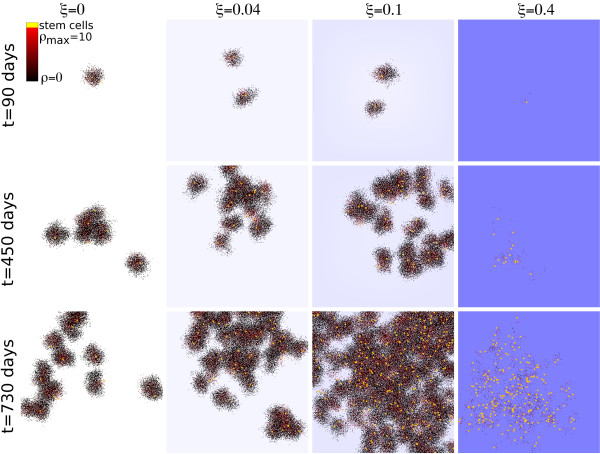
**Immune system modulated morphological evolution.** Representative sample simulations of tumor morphologies after 90, 450 and 730 days for different immune reactant source strengths ξ. Shaded background represents immune system strength with darker blue representing a stronger local immune reactant concentration.

As described in detail elsewhere [13], heterogeneous tumors comprised of cancer stem cells and non-stem progenitors grow as conglomerates of ‘self-metastases’ – i.e. of cancer stem cells seeding the tumor periphery – which are rapidly surrounded and inhibited by their own non-stem progeny. By contrast, the relaxation of spatial constraints made possible by cell death [26], allows for cancer stem cell migration to these now less dense regions and the potential expansion of the cancer stem cell pool. Consequently, a low cytotoxic immune response does not reduce the tumor burden but instead promotes self-metastatic tumor growth (Figure 4). Only with a sufficiently high immune reaction can a reduction in tumor size be observed, but this comes at the expense of enriching for cancer stem cells. Under these conditions, emulated by simulation once ξ≈0.4, dense tumor clusters disappear and become replaced by an unconnected patched morphology as previously observed in other cellular automaton studies [19]. In the patient setting, the residual and now isolated cancer stem cells will inevitably go on to drive the growth of a more resistant malignant tumor.

### Early and late effects of an adaptive immune response

**Figure 5:**
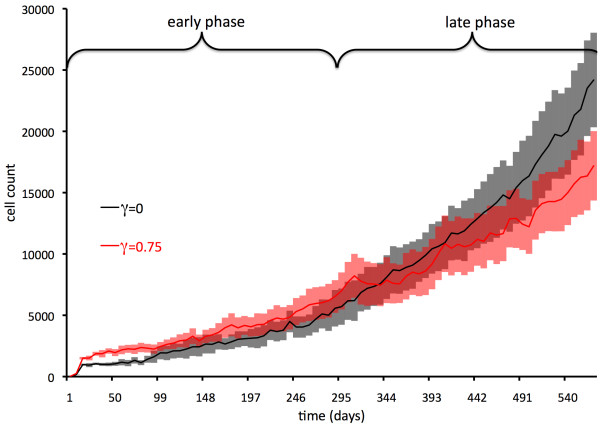
**Early and late function of the immune system on neoplasia.** Dual function of the immune response in relation to tumor progression; an early minimal reaction that promotes, and a late strong reaction that inhibits (after Prehn, 1972). Shown are averages of 10 independent simulations and standard error.

We now consider an immune reaction proportional to the size of the growing tumor. For this, we assume tumor cells emit a chemical signal *m* that diffuses (with comparable diffusion rate d_m_ = 10^-9^ cm^2^s^-1^) into the domain Ω and triggers an immune response at the boundary ${c|}_{\partial \Omega }$ according to the strength of the signal there. Specifically,

(3) $$\begin{array}{l} \begin{array}{cc} \frac{\partial m}{\partial t}=\overset{\text{production}}{\overset{︷}{\gamma P(x,y,t)}}+\overset{\text{diffusion}}{\overset{︷}{d_{m}\nabla^{2}m}}-\overset{\text{decay}}{\overset{︷}{\beta_{m}m}}, & \;\gamma \in [0,1] \end{array}\\ c{|}_{\partial \Omega }=m{|}_{\partial \Omega }\text{,} \end{array}$$

where *P*(*xyt*) represents the occupation status in the discrete cell layer (1 if a cell is present at (*xy*) at time *t* and 0 otherwise). What is seen after simulation is that a small tumor cluster triggers only a low response, whereas a large conglomerate of self-metastases induces a strong immune reaction. Tumor growth dynamics (Figure 5) feature a promotion of tumor growth early on while the immune reaction is low, followed by a late strong reaction that is inhibiting – the early and late function of an initially weak and later strong immune system response to a growing tumor, another effect hypothesized by Prehn [2].

## Discussion

We presented a cellular automaton model of heterogeneous tumor growth and the impact of an induced immune response on tumor dynamics. Intrinsically, without an immune response, a heterogeneous tumor population comprised of cancer stem cells and non-stem progenitors grows as conglomerates of self-metastases [13,14]. This morphological phenomenon results from the interplay of cell proliferation, cell migration and cell death. With increasing cell death intra-tumoral spatial inhibitions are loosened, which in turn enable cancer stem cell cycling and thus, counter-intuitively, tumor progression. Focusing only on the cytotoxic function of the immune system we were able to observe all immunoediting roles of the immune system: immune promotion at weak immune responses, immunoinhibition at strong immune responses, and immunoselection at all levels. Simulations of our model support a hypothesis previously put forward by Prehn [2,8-10] that comparable tumor sizes can be observed for weak and strong immune reactions (either side of the peak in Figures 1 and 3). Our model augments these studies by highlighting the different tumor compositions expected, including a malignant enrichment in cancer stem cells following a strong immune response. We conclude that tumors that progress to clinical presentation, particularly after strong immune responses, are likely to be heavily enriched in cancer stem cells. Moreover, when the immune system selection force is removed, the initial ratio of cancer stem cells to non-stem cells is re-established, showing that long-term cancer stem cell enrichment requires continuous dynamic maintenance. We propose more generally that a stem-cell-expansive influence may take the form of anything that encourages morphological fingering. Beyond immune response, this could include cell death, or even growth within restricted thin channels, as might be expected e.g. during invasion of host tissue.

## Competing interests

The authors declare that they have no competing interests.

## Authors’ contributions

HE conceived of the study, participated in the study coordination, executed the model simulations, analyzed the data, and drafted the manuscript. LH and PH conceived of the study, participated in the study coordination and helped draft the manuscript. All authors read and approved the final manuscript.
